# Electric plasma guided with ultrasonic fields

**DOI:** 10.1126/sciadv.adp0686

**Published:** 2025-02-05

**Authors:** Josu Irisarri, Iñigo Ezcurdia, Naroa Iriarte, Marika Sirkka, Dmitry Nikolaev, Joni Mäkinen, Alexander Martinez-Marchese, Denys Iablonskyi, Ari Salmi, Asier Marzo

**Affiliations:** ^1^ UpnaLab, Public University of Navarre, Pamplona 31006, Spain.; ^2^ Electronics Research Laboratory, University of Helsinki, Helsinki FIN-00014, Finland.; ^3^ MSAM Lab, University of Waterloo, Waterloo, ON N2G 4X8, Canada.

## Abstract

Electric plasma forms sparks in midair that transfer electrical current. This current can power high-voltage electronics, kill bacteria, produce tactile sensations, or be used for welding. However, the formation of the spark is chaotic and hard to control. Laser pulses can guide discharges but require high power and are disruptive and cumbersome to control. Here, we show that ultrasonic fields can guide plasma sparks, even around obstacles. The ultrasonic beams can be directed dynamically and within milliseconds, enabling precise, nondangerous, and fast control of high-voltage sparks. This phenomenon can be used for applications in high-voltage switching and plasma treatments.

## Teaser

Dynamic ultrasonic beams can guide electric sparks with millimeter accuracy within milliseconds and around complex trajectories.

## INTRODUCTION

The formation of high-voltage sparks upon electric breakout produces branches of plasma in midair. High-voltage sparks on the centimeter scale can be used for ignition (*1*), tactile sensations (*2*), killing germs (*3*), surface treatments (*4*), welding (*5*), milling (*6*) or switching high-voltage electronics (*7*). Controlling the path of electric discharges would markedly improve those applications, e.g., from just targeting a single point to creating two-dimensional (2D) dynamic patterns or from following a chaotic path to the spark being guided around obstacles.

Some physical principles can be used to affect electric sparks. Alpha particles (*8*), x-rays (*9*), or gamma radiation (*10*) can ionize air along its path and initiate the formation of a spark. However, using these highly energetic particles or waves is not feasible for the control of sparks, given the difficulty to generate them along controlled paths; also, they entail dangers for living organisms. Microwave radiation can also facilitate the formation of a spark (*11*) but not along a controlled path.

The flow of noble gases along an electric plasma extends its reach and reduces its temperature (*12*). This is referred to as cold plasma (*13*) and has numerous applications in food and biomaterials processing. However, it requires a constant flow of a noble gas and, although it extends the reach and straightness of the plasma, it cannot dynamically control it without valves and other mechanical components.

Nakane *et al.* (*14*, *15*) showed in 1987 that a corona discharge gets broadened when an acoustic antinode from a standing wave is applied into it. Balek *et al.* (*16*) observed a similar effect in 2006. The widened area of corona discharge increased ozone generation (*17*) and current transfer (*18*). The phenomenon becomes stronger when airflow is applied (*19*).

Discharge guided via laser pulses uses a high-power laser to create a plasma that leads to a heated region of lower-density gas, thereby providing a preferred path for discharge (*20*). Pulsed lasers can be used to guide centimeter-sized sparks (*7*, *20*), and Airy beams can even bend a spark around an obstacle (*21*). Laser-guided discharge is the only method for finely guiding electric discharges in midair; however, using high-power lasers requires careful control of the delays between the laser pulses and the discharge, the equipment is cumbersome, and the laser beam itself can damage surfaces or electrodes.

In this work, we report the use of dynamic ultrasonic beams to guide the electric plasma with millimeter accuracy and millisecond response time and around complex trajectories (see movie S1).

## RESULTS

### Controlled guidance

**Fig. 1.**
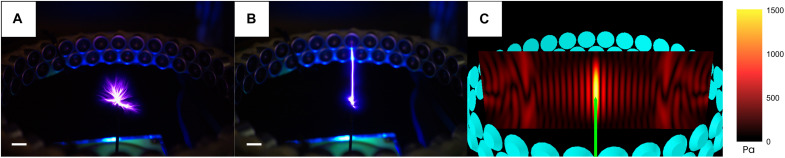
Ultrasonic field guiding electric plasma. (**A**) Plasma spark without the ultrasonic field applied. (**B**) Plasma spark with the ultrasonic field. (**C**) Amplitude of the acoustic field (electrode in green). Scale bars, 1 cm.

The electric spark follows the generated ultrasonic focal point (Fig. 1). Therefore, if the focal point is tilted, the spark is guided toward different positions. Two tilting strategies are used: mechanical and array control. Mechanical tilting is performed by rotating the ultrasonic emitters, whereas in array control, the emission of the emitters is adjusted electronically without mechanical actuation. A setup of two concentric rings was used to mechanically tilt the ultrasonic focal point with 2 degrees of freedom (fig. S1).

**Fig. 2.**
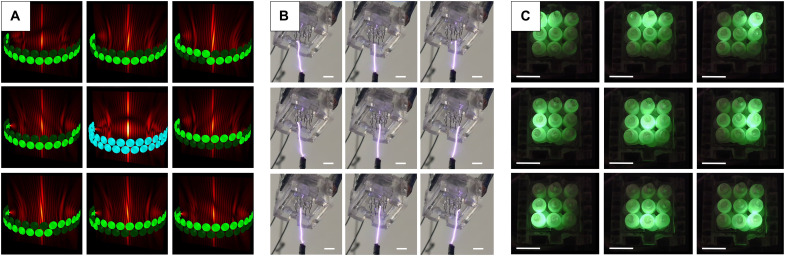
Activating specific neon bulbs by targeting a 3 by 3 array. (**A**) Ultrasonic emitters and generated amplitude field. (**B**) Plasma spark targeting individual electrodes. (**C**) Illumination of the corresponding neon bulbs. Scale bars, 1 cm.

The plasma spark can also be tilted with dynamic acoustic fields produced from electronic control of the ultrasonic emitters. In fig. S2, by activating half of the array at different rings, the focal point gets tilted, thus tilting the guided spark. In Fig. 2, we show electronic control on the azimuth and inclination of the spark to target individual electrodes in a 3 by 3 array for energizing specific neon light bulbs. The maximum clean switching speed was 100 ms, and it was possible to switch faster, but the spark momentarily hits other electrodes.

### Target materials

The ultrasonic guidance of the spark allows it to hit targets of different materials. In fig. S3, the spark is guided toward flat sheets of both nonconductive (acrylic and paper) and conductive materials (paper with graphite, perfboard, and tinfoil). We note that when the spark is not guided with ultrasound, it does not hit some materials or hits them at varying positions. With the ultrasonic guidance, both conductive and nonconductive materials can be targeted at a fixed spot.

### Complex fields: Avoiding an obstacle

**Fig. 3.**
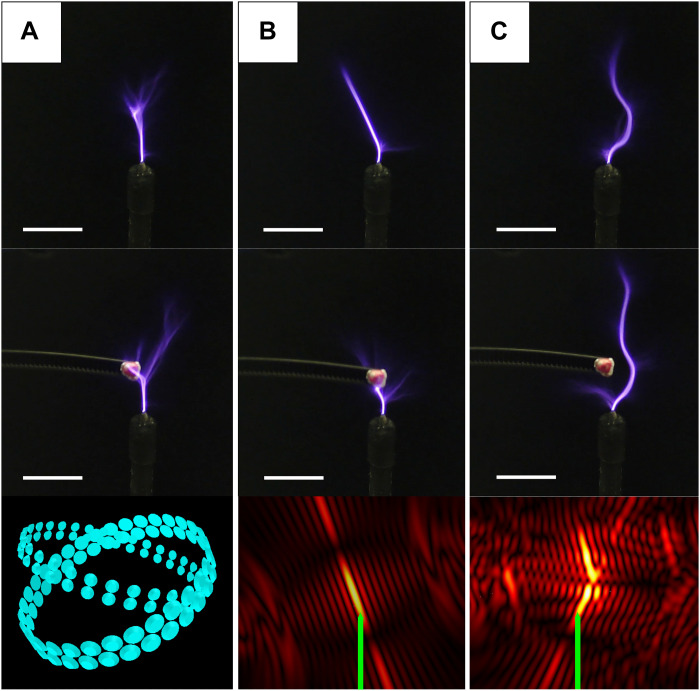
Guidance of the spark along a curved acoustic field created with two focal points coming from two rings of emitters. (**A**) No ultrasound. (**B**) Only one focal point. (**C**) Curved field created with two intersecting focal points. The top row shows the spark without the presence of the obstacle, middle row with a popcorn kernel, and bottom the amplitude field. The electrode is marked in green. Scale bars, 1 cm.

Acoustic fields are not limited to straight beams from a focal point. Here, we test complex fields such as curved focal points. In fig. S4, two focal points are intersected to create a bent focal point that guides the spark along a sharp turn. In Fig. 3, two focal points are aligned at different angles to create a curved spark that can avoid an obstacle and continue upward.

### Time dynamics

**Fig. 4.**
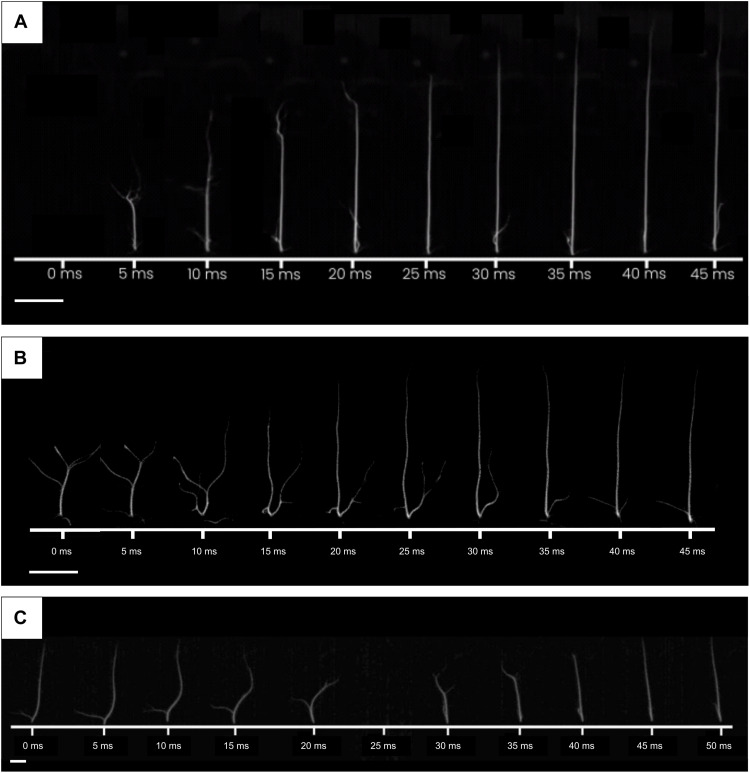
Spark being guided at different time stamps. (**A**) The spark is switched on when the ultrasonic field is already on. (**B**) The spark is already on, and the ultrasonic field is switched on at *t* = 0. (**C**) The spark is tilted right to left with dynamic array control. Scale bars, 1 cm.

The time that it takes for the ultrasonic field to guide the plasma spark has been measured under different conditions. If the ultrasonic field is already active and the spark is switched on, the spark takes 15 ms to be guided and 30 ms to reach full length (Fig. 4A). When the ultrasonic field is activated upon an existing spark, the average time until the spark moves to the required position is 35 ms (Fig. 4B). When the focal point was tilted right/left at 10°, the stabilization time was 40 ms (Fig. 4C), and it can be seen that the tilting starts from the lower part of the spark.

### Mechanism of the guidance

The air in the vicinity of the plasma streamer is heated up, which is the result of collisions between the charged particles accelerated by an electric field and other neutral air molecules (*22*). The heated air has a different acoustic impedance than the surrounding air since they have different temperatures and, thus, densities and traveling speed of sound. This leads to the appearance of an acoustic radiation force that pushes the hot air into the antinodes. This phenomenon has been reported between different gasses, where the gas with less acoustic impedance gets trapped in the antinodes of a standing wave (*23*).

**Fig. 5.**
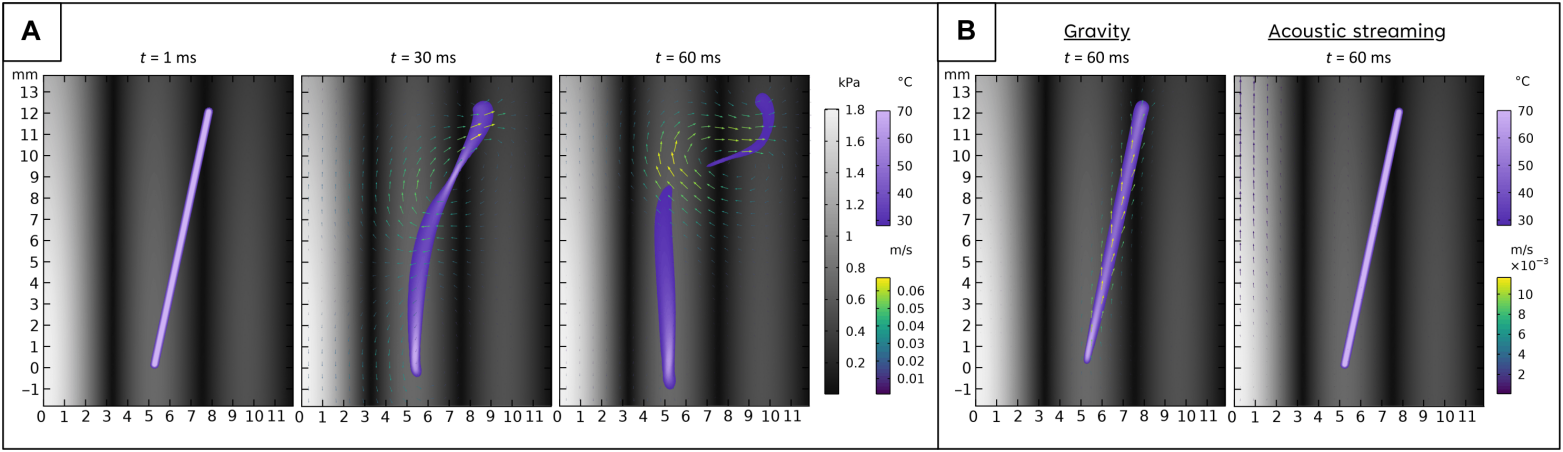
Simulations of lower-density hot air filaments inside an acoustic field. (**A**) Distributions of temperature, air velocity, and acoustic pressure at 1, 30, and 60 ms. (**B**) Only gravity and acoustic streaming are applied.

Simulations of the trapping and straightening of lower-density hot air are presented in Fig. 5A. In Fig. 5B, only gravity and streaming are simulated, the guiding effect is not observed, and thus, we suggest that the main force on the hot air is due to the acoustic radiation force arising from the presence of acoustic contrast between heated lower-density air and ambient air. More simulations are shown in figs. S5 and S6 and movie S3. They show that lower-density hot air regions will be guided along the high-amplitude regions (antinodes).

The heated and lower-density region of air can be seen experimentally in movie S4 and fig. S7. A Schlieren setup was used to visualize air at different temperatures and densities. Without the application of ultrasound, the spark creates a turbulent flow of hot air that goes upward. When the ultrasonic field is applied, the lower-density hot air region is constrained along a straight line in the high-amplitude area of the focal point.

**Fig. 6.**
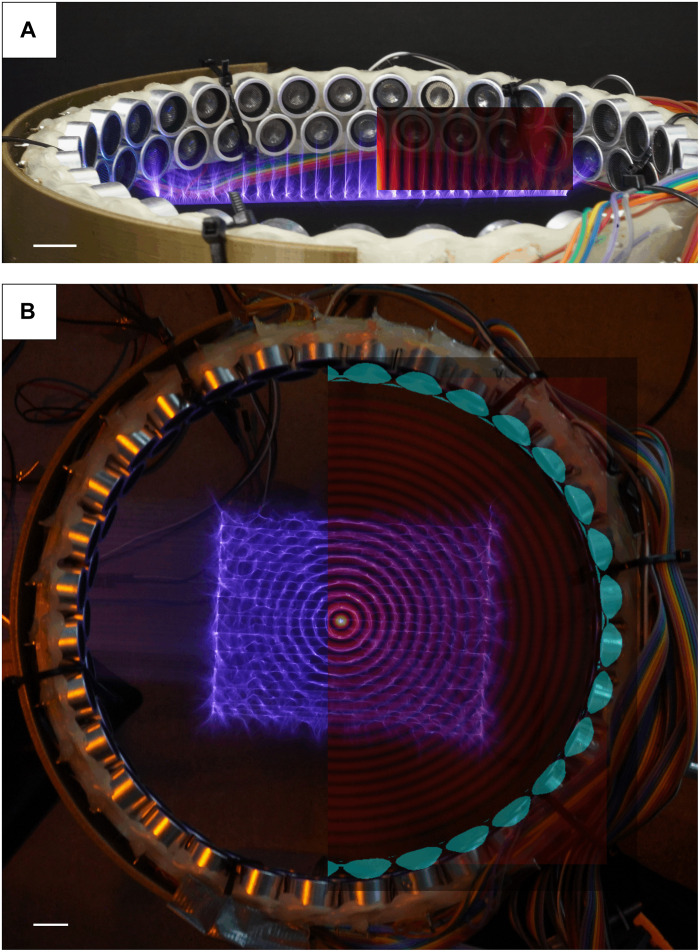
Long-exposure picture of the electric spark while the Tesla coil is translated inside an ultrasonic ring. (**A**) Side view while the coil is translated in one dimension. (**B**) Top view while the coil is scanned in two dimensions using a CNC stage. In the right halves of the pictures, the simulated amplitude fields have been overlaid. 1D translation is 5 s, and the 2D scanning time is 20 s. Scale bars, 1 cm.

The heated regions of lower-density air provide a preferred path for discharge, as described before for guidance using pulsed lasers (*20*). Experimentally, it is apparent that the discharges follow the high-amplitude regions where the heated and lower-density air concentrates (Fig. 6 and figs. S8 and S9).

We neglected the direct effect of the ultrasonic waves on the breakout voltage because it fluctuates over time, therefore yielding no substantial change when averaged over the wave period. Furthermore, the effect on the breakout voltage from lower-density heated air is an order of magnitude larger than that from pressure fluctuation. In our experiments, we used peak sound pressures of 1500 Pa, which, according to Paschen’s law (*24*), affect breakout as ±460 V/cm [*A* = 11.25 (Pa m)^−1^, *B* = 273.75 V/(Pa m), γ = 0.01, and *p*_0_ = 101325 Pa]. On the other hand, if the heated and lower-density region of air is at 70°C (measured from the experiments) and the ambient air at 25°C, the breakout voltage is decreased by 4091 V/cm [calculated using (*24*)].

In summary, the electric spark heats up the air; this heated air expands, thus decreasing in density; the heated lower-density air is shaped by the ultrasonic field that pushes it to the high-amplitude regions; and the region of lower-density air is a preferred discharge path. This is in line with laser-guided discharge (*20*), but the heated lower-density region of air is created by the spark itself and shaped by the ultrasonic field.

## DISCUSSION

The electric spark used in the experiments is an alternating current of 2.3 MHz with a square amplitude modulation ranging from 50 to 4000 Hz (figs. S10 and S11). The ultrasonic field had an effect on a dc spark, but it was not capable of guiding it. We tested a spark with a gap of 2 cm at 60 kV (see fig. S12); other dc sparks with smaller gaps using 4 kV (EMCO cube) or 11 kV (High Voltage, Dekaim) could not be guided either. We think that this is due to the ionic wind (*25*) created between the pair of electrodes. We consider this a limitation, yet the applications described throughout this paper operate with ac sparks.

The ultrasonic field was shown to guide an electric spark to target different spots, increase its range, and allow it to hit different types of materials. High-voltage switching seems the main application, but other applications are highlighted, for instance, patterning bacteria in petri dishes (fig. S13) or contactless haptic displays that do not require the user to wear any device (movie S2 and fig. S14). Another traditional use of sparks is the generation of shock waves (*26*); ultrasound could guide the spark along complex trajectories to control the waveform of its shock wave.

Using ultrasonic fields to guide electric sparks provides unique features when compared with laser-guided discharge. It is simpler and safer to use ultrasonic fields in the order of 1500 Pa than high-power pulsed lasers. The laser can damage people’s sight or have an effect on the treated samples and electrodes. In general, pulsed lasers are more expensive and cumbersome than the ultrasonic generators.

Laser-guided discharge requires accurate timing between the optical pulses and electric discharges, whereas the ultrasonic devices do not need synchronization with the electric discharge. The ultrasonic guidance can operate continuously (as shown in movie S1), and laser guidance has only been shown for individual pulses.

Ultrasonically guided sparks are uncontrolled in the first milliseconds after the activation (Fig. 4B), whereas a laser-guided discharge is controlled from the first discharge. This is because in shaping with ultrasound, the heated lower-density region of air takes some milliseconds (see movie S4), whereas in laser guiding, the region of hot air is directly generated by the laser before the first discharge. Future research can focus on releasing hot air that gets shaped by the ultrasonic field before the first spark; therefore, the first sparks would be guided.

With ultrasound, we have guided ac rather than dc as is the case in laser-guided discharges. ac has the advantage of not requiring a pair of electrodes between the discharge or having to ground the target; we note however that it can limit some applications related to high-power electronics that only operate with dc.

We have shown the phenomenon of using ultrasonic fields to guide an electric plasma spark. The accurate and fast-forming patterns generated by the ultrasonic fields allow for the redirection and shaping of electric sparks in unprecedented ways, opening up applications for high-voltage switching, germs patterning, haptics, ignition, or welding.

## MATERIALS AND METHODS

### Electric discharge generation

A Tesla coil (Yosoo Health Gear) is used to generate an electric discharge with a carrier frequency of 2.4 MHz. An amplitude modulation ranging from 50 Hz to 5 kHz and duty cycle from 40 to 50% are applied to the Tesla coil using a signal generator. In fig. S11, the reaction time of the spark to the ultrasound at different modulation frequencies can be seen. The Tesla coil was powered with a regulated power supply between 20 and 45 V, with an average consumption of 1A. If not indicated, the parameters are a modulation of 2.5 kHz, 50% duty cycle, and an input of 48 V.

### Ultrasonic array

An ultrasonic array with two rings was used: diameter of 17 cm and 32 emitters per ring operating at 40 kHz (MSO-A1640H10T, Manorshi Electronics Co., Ltd., China). The emitters were driven with an Ultraino board (*27*) powered at 15 V. The control software was running on a PC.

### High-speed footage and timing

A high-speed camera MIRO C211 Monochrome equipped with a Fujinon 1:16/35-mm lens was used to record at 10,000 frames/s for 200-μs exposure time. A light-emitting diode was attached to the ultrasonic ring to determine the reaction times so that it would switch on when the ultrasonic array is on. The time that it takes for the ultrasound to reach from the emitters into the spark is neglected (below 0.3 ms). A picture of the Tesla coil, ultrasonic array, high-speed camera, and other equipment can be seen in fig. S15.

### Acoustic amplitude simulations

To calculate the complex pressure amplitude radiated by a single emitter, we used the approximation of the far-field radiation of a baffled piston (*28*). The field generated by the array of emitters is calculated as a superposition of the radiation of *N* emitters

$$p\left(r\right)=\sum_{i=1}^{N}p_{0}\frac{e^{{\mathrm{ikr}}_{i}}}{r_{i}}\frac{2J_{1}\left(\mathrm{ka}\;\text{sin}\;\theta_{i}\right)}{\mathrm{ka}\;\text{sin}\;\theta_{i}}$$ (1)

where $p_{0}$ is the pressure amplitude of one individual emitter at a distance of 1 m in pascal when the emitter is excited by a square signal with an amplitude of 1 V. $p_{0}=0.72$ for the used emitters. a=4.5 mm is the radius of the emitters, and $r_{i}$ is the vector pointing from the emitter i to the point in space with radius vector r, $r_{i}=|r_{i}|$. $\theta_{i}$ is the angle between the normal of an emitter and the vector $r_{i}$.

### Trapping of hot air simulations

The process of the trapping of a hot area was dynamically simulated using the finite element method. The model was built using Comsol Multiphysics (COMSOL AB, Stockholm, Sweden). An axisymmetric model was created, representing an experimental ring setup with disk-shaped transducers (radius a=4.5 mm) located on the surface of the ring. The acoustic field was simulated using the pressure acoustics module in frequency domain, and acoustic streaming was simulated with the laminar flow module. The physics were coupled with the acoustics module using the acoustic streaming domain coupling. The effect of hot air areas was added using the heat transfer physics module, together with the nonisothermal flow coupling. The domain edges were modeled as an open boundary. The oscillatory velocity of the emitters was set in the same phase and with amplitude such that the array generated the pressure at the center of the array of the same amplitude that was measured in the experiment. As an initial condition of the problem, the hot air region was defined as a straight inclined filamentous region with a diameter of 0.3 mm, a length of 12.5 mm, and a temperature of 70°C, coinciding with the measured temperature of an electric spark.

### Schlieren setup

A reflective Schlieren setup was used with a first surface telescope mirror of 350 mm in diameter and 2.5 m in focal length. The mirror and subject to study were placed at one side, whereas the light source (a white light-emitting diode covered with a pierced piece of black tape), razor knife (covering half of the focal point), and camera were placed at the other side.

## References

[1] J. D. Dale, M. D. Checkel, P. R. Smy, Application of high energy ignition systems to engines. Prog. Energy Combust. Sci. 23, 379–398 (1997).

[2] D. Spelmezan, D. R. Sahoo, S. Subramanian, “Sparkle: Hover feedback with touchable electric arcs,” in *Proceedings of the 2017 CHI Conference on Human Factors in Computing Systems* (2017), pp. 3705–3717.

[3] S. A. Mir, M. A. Shah, M. M. Mir, Understanding the role of plasma technology in food industry. Food Bioproc. Tech. 9, 734–750 (2016).

[4] Y. Tsuchiya, K. Akutu, A. Iwata, Surface modification of polymeric materials by atmospheric plasma treatment. Prog. Org. Coat. 34, 100–107 (1998).

[5] R. K. Tyagi, R. S. Pandey, A. K. Kumar, K. K. Srivastava, Effect of electric and magnetic field on welding parameters in plasma welding. Int. J. Eng. Sci. Technol. 3, 168–176 (2012).

[6] A. Calka, D. Wexler, Mechanical milling assisted by electrical discharge. Nature 419, 147–151 (2002). 12226660 10.1038/nature00985

[7] B. M. Luther, L. Furfaro, A. Klix, J. J. Rocca, Femtosecond laser triggering of a sub-100 picosecond jitter high-voltage spark gap. Appl. Phys. Lett. 79, 3248–3250 (2001).

[8] D. Azimi-Garakani, “Spark counter for alpha particle registration,” in *Proceedings of the International Workshop on Radon Monitoring in Radioprotection, Environmental Radioactivity and Earth Sciences* (1990), pp. 164–170.

[9] S. Frankel, V. Highland, T. Sloan, O. Van Dyck, W. Wales, Observation of x-rays from spark discharges in a spark chamber. Nucl. Instrum. Methods 44, 345–348 (1966).

[10] H. Helmken, G. Fazio, Vidicon spark chamber detector for gamma-ray astronomy. IEEE Trans. Nucl. Sci. 13, 486–492 (1966).

[11] H. Wu, Z. Wang, C. Liu, J. Xu, X. Cheng, J.-Y. Chen, X. Zhang, Two enhancements in microwave-assisted spark ignition and their causes. Combust. Flame 252, 112744 (2023).

[12] M. Laroussi, T. Akan, Arc-free atmospheric pressure cold plasma jets: A review. Plasma Processes Polym. 4, 777–788 (2007).

[13] S. K. Pankaj, K. M. Keener, Cold plasma: Background, applications and current trends. Curr. Opin. Food Sci. 16, 49–52 (2017).

[14] T. Nakane, T. Hirata, K. Seya, Behavior of electric discharge in ultrasonic field. Jpn. J. Appl. Phys. 26, 203 (1987).

[15] T. Nakane, Discharge phenomenon in a high-intensity acoustic standing wave field. IEEE Trans. Plasma Sci. 33, 356–357 (2005).

[16] R. Bálek, S. Pekárek, Z. Bartáková, Power ultrasound interaction with dc atmospheric pressure electrical discharge. Ultrasonics 44, e549–e553 (2006). 16793088 10.1016/j.ultras.2006.05.121

[17] R. Bálek, S. Pekárek, Z. Bartáková, Ultrasonic resonator with electrical discharge cell for ozone generation. Ultrasonics 46, 227–234 (2007). 17395233 10.1016/j.ultras.2007.01.011

[18] R. Bálek, M. Červenka, S. Pekárek, Acoustic field effects on a negative corona discharge. Plasma Sources Sci. Technol. 23, 035005 (2014).

[19] S. Pekárek, R. Bálek, Ultrasound and airflow induced thermal instability suppression of DC corona discharge: An experimental study. Plasma Sources Sci. Technol. 15, 52–58 (2006).

[20] S. Tzortzakis, B. Prade, M. Franco, A. Mysyrowicz, S. Hüller, P. Mora, Femtosecond laser-guided electric discharge in air. Phys. Rev. E 64, 057401 (2001). 10.1103/PhysRevE.64.057401 11736149

[21] M. Clerici, Y. Hu, P. Lassonde, C. Milián, A. Couairon, D. N. Christodoulides, Z. Chen, L. Razzari, F. Vidal, F. Légaré, D. Faccio, R. Morandotti, Laser-assisted guiding of electric discharges around objects. Sci. Adv. 1, e1400111 (2015). 26601188 10.1126/sciadv.1400111 PMC4640611

[22] N. Popov, Fast gas heating in a nitrogen–oxygen discharge plasma: I. Kinetic mechanism. J. Phys. D Appl. Phys. 44, 285201 (2011).

[23] R. Tuckermann, B. Neidhart, E. G. Lierke, S. Bauerecker, Trapping of heavy gases in stationary ultrasonic fields. Chem. Phys. Lett. 363, 349–354 (2002).

[24] G. Galli, H. Hamrita, C. Jammes, M. J. Kirkpatrick, E. Odic, P. Dessante, P. Molinié, Paschen’s law in extreme pressure and temperature conditions. IEEE Trans. Plasma Sci. 47, 1641–1647 (2019).

[25] E. Moreau, P. Audier, T. Orriere, N. Benard, Electrohydrodynamic gas flow in a positive corona discharge. J. Appl. Phys. 125, 133303 (2019).

[26] J. W. Mackersie, I. V. Timoshkin, S. J. MacGregor, Generation of high-power ultrasound by spark discharges in water. IEEE Trans. Plasma Sci. 33, 1715–1724 (2005).

[27] A. Marzo, T. Corkett, B. W. Drinkwater, Ultraino: An open phased-array system for narrowband airborne ultrasound transmission. IEEE Trans. Ultrason. Ferroelectr. Freq. Control 65, 102–111 (2018). 29283352 10.1109/TUFFC.2017.2769399

[28] G. S. Kino, *Acoustic Waves: Devices, Imaging, and Analog Signal Processing* (Prentice Hall, 1987).

