# Supplementary material for: Electric plasma guided with ultrasonic fields
Source: Sci Adv. 2025 Feb 5;11(6):eadp0686. doi: 10.1126/sciadv.adp0686 (PMC11797549; doi:10.1126/sciadv.adp0686)
Supplement: Supplementary file 1 — Figs. S1 to S15 Legends for movies S1 to S4 [file sciadv.adp0686_sm.pdf]

Science Advances
AAAS

Supplementary Materials for

Electric plasma guided with ultrasonic fields

Josu Irisarri *et al.*

Corresponding author: Asier Marzo, asier.marzo@unavarra.es

Sci. Adv. **11**, eadp0686 (2025)
DOI: 10.1126/sciadv.adp0686

The PDF file includes:

Figs. S1 to S15
Legends for movies S1 to S4

Other Supplementary Material for this manuscript includes the following:

Movies S1 to S4

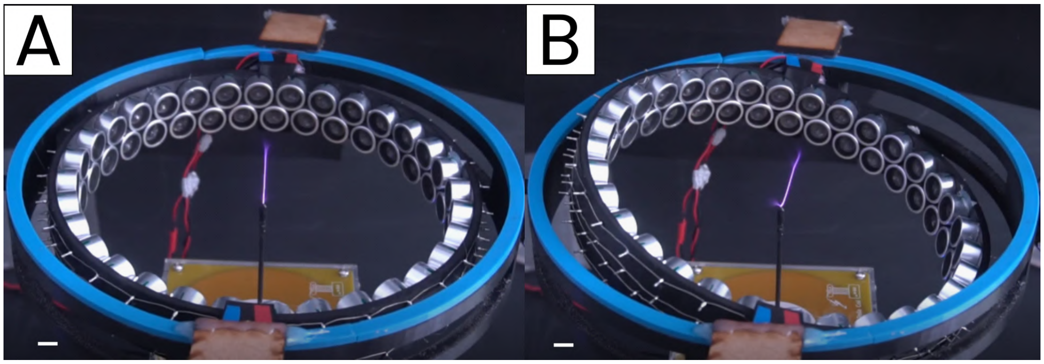

Fig. S1: Plasma spark guidance with a mechanical tilting of the array of ultrasonic emitters. A) No tilting. B) Tilting 17° to the right. Scale bars are 1 cm.

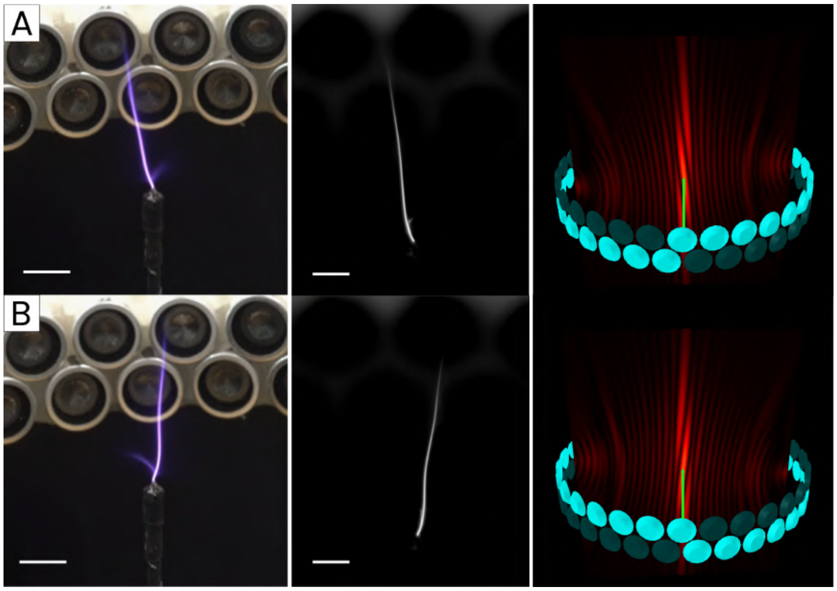

Fig. S2: Control of the inclination of the spark by electronically adjusting the ultrasonic emission. A) Tilting to the left. B) Tilting to the right. Left column) real photos, middle column) low-exposure pictures. right column) simulations of the amplitude field, the electrode is marked in green. Scale bars are 1 cm.

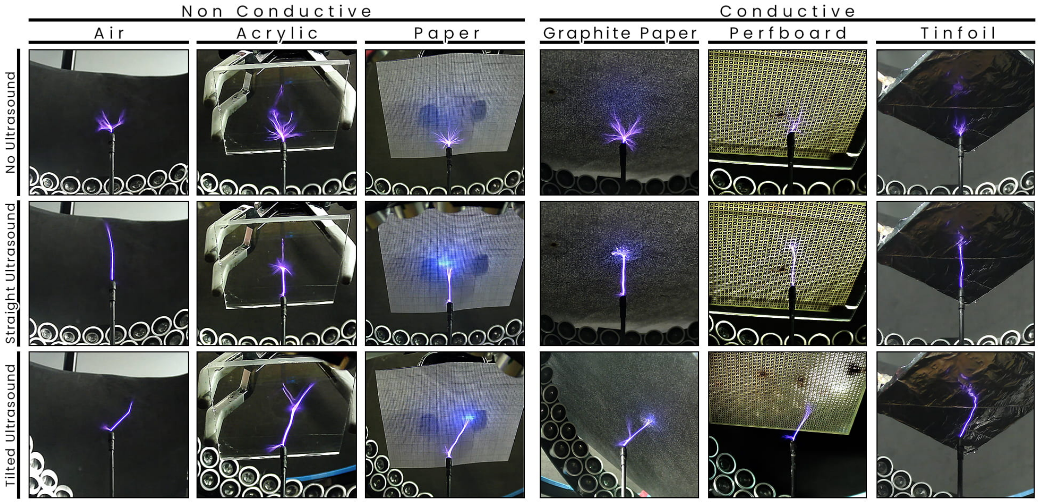

Fig. S3: Plasma spark being guided to hit different materials. Top row) No ultrasound. Middle row) With a focal point parallel to the electrode. Bottom row) with a tilted focal point.

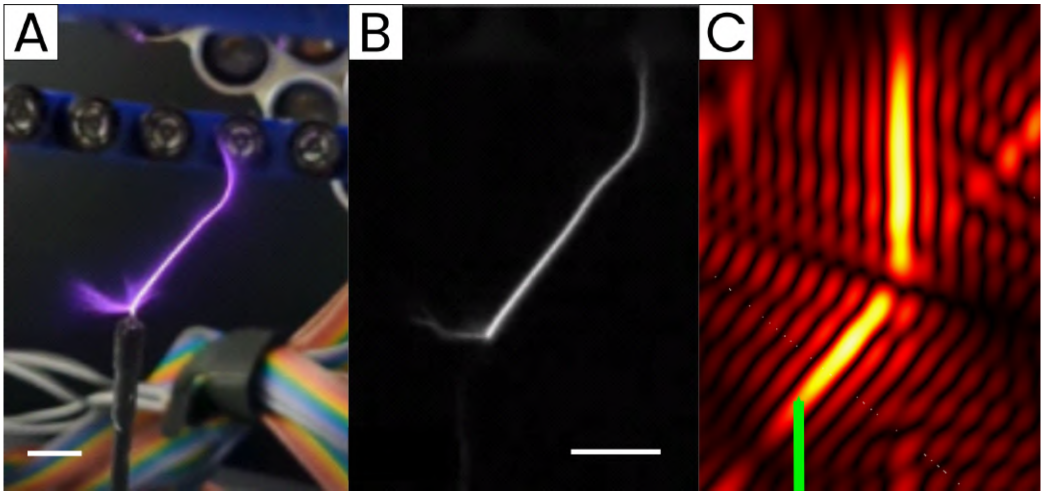

Fig. S4: Guidance of the spark along two intersecting focal points. A) Regular pictures. B) Low-exposure picture. C) Acoustic simulation of the amplitude field, the electrode is marked in green. Scale bars are 1cm.

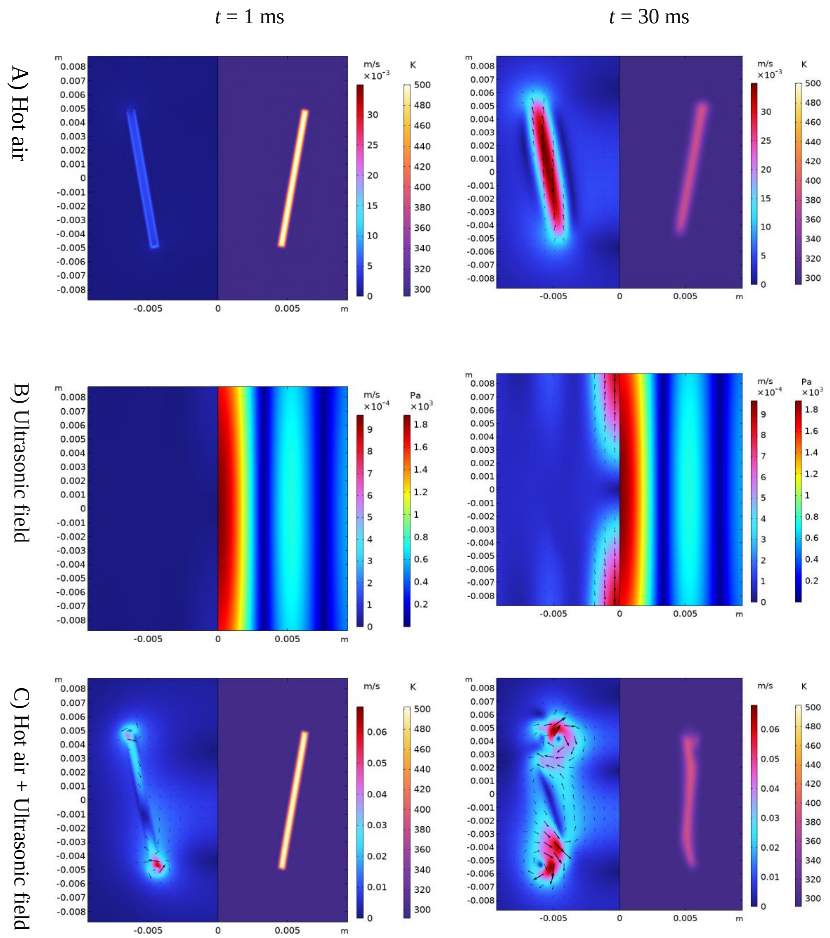

Fig. S5: Straightening of a hot-air region under the ultrasonic field from t = 1 ms (left column) and in t = 30 ms (right column). (A) Just hot air: air flow (left) and temperature distribution (right). (B) Just ultrasonic field: Acoustic streaming (left) and pressure field (right). (C) Hot-air region inside the influence of an ultrasonic focal point: air flow (left) and temperature distribution (right). The arrows indicate the direction of air streaming.

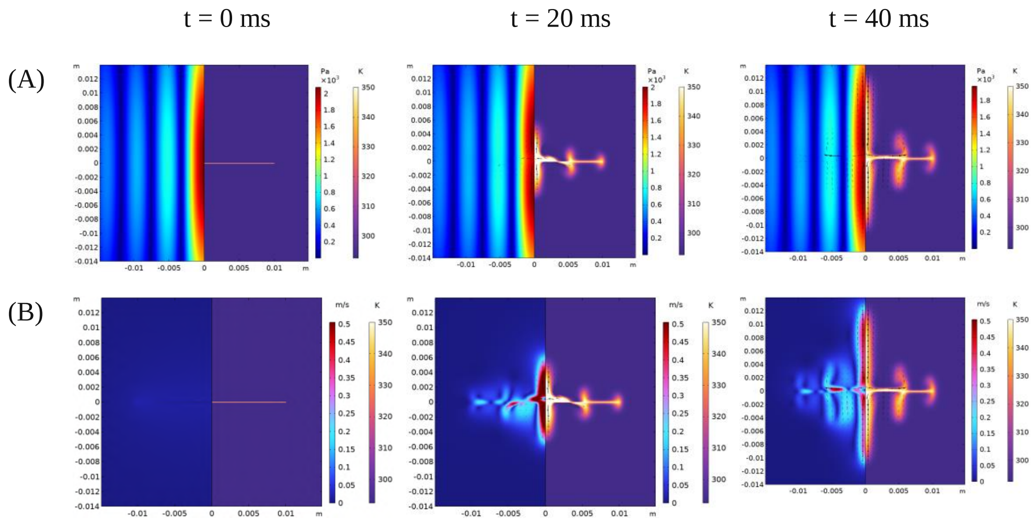

Fig. S6: Horizontal hot-air region getting trapped on the different anti-nodes. From t = 0 ms (left column), to t = 40 ms (right column). (A) Pressure field (left) and temperature distribution (right). (B) Acoustic streaming (left) and temperature distribution (right).

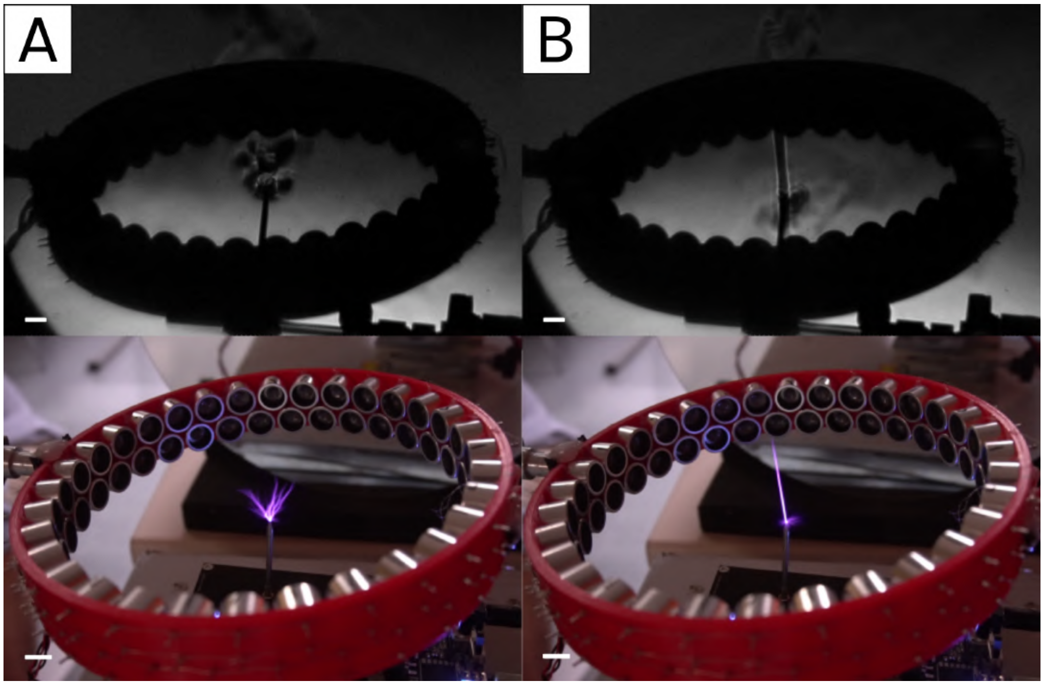

Fig. S7: Schlieren visualization of air temperature and density generated by a spark. A) without ultrasound. B) guided by ultrasound. Scale bars are 1 cm.

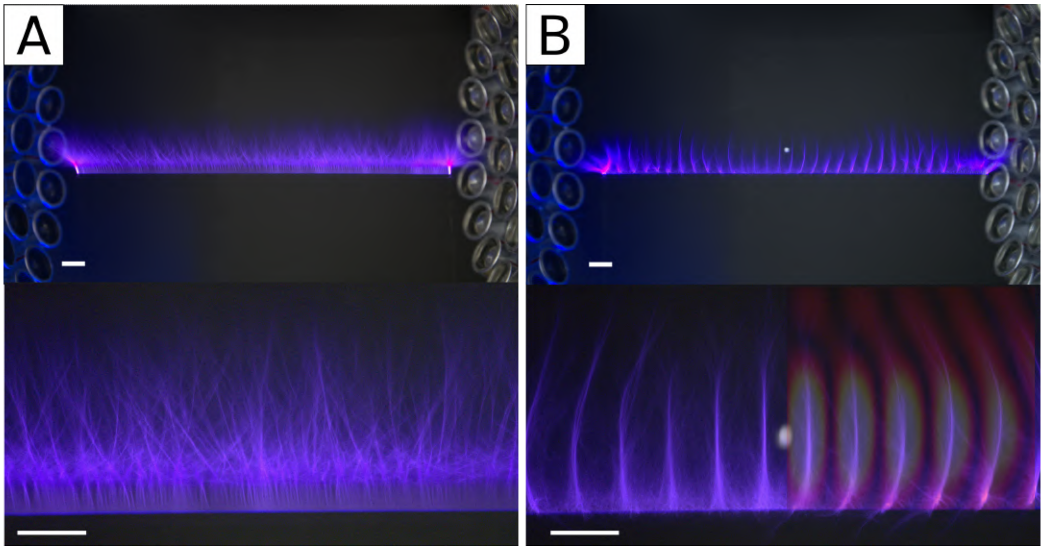

Fig. S8: Long-exposure picture of the plasma spark while the Tesla coil is translated from left to right inside a standing wave levitator. A) No acoustic field. B) Acoustic field is on. The amplitude field has been overlaid in the right half of the picture. We note that the spark follows the high-amplitude regions (antinodes) whereas a particle remains trapped in the node. Scale bars are 1 cm. Translation time is 5 seconds.

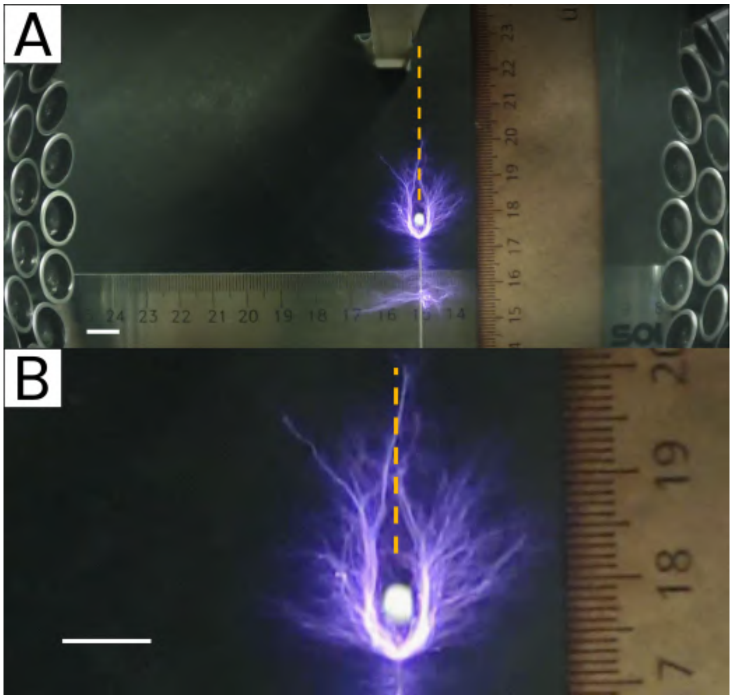

Fig. S9: Spark in a standing wave. A particle is trapped in the node whereas the spark is guided along the anti-nodes (high-pressure regions). The yellow line marks the node location. Scale bars are 1 cm.

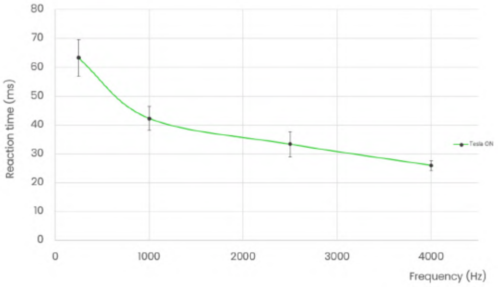

Fig. S10: Time that takes the guided spark to reach full length upon its activa-tion under an ultrasonic field. Time depending on the modulation frequency of the spark. Plasma spark guided at 2500 Hz. Experiments were repeated 4 times. Scale bar is 1 cm.

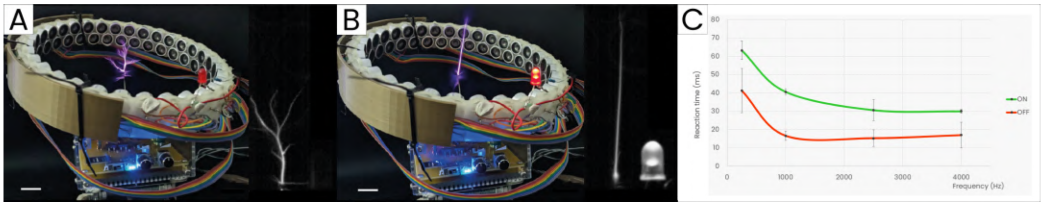

Fig. S11: Time to guide or stop guiding an existing spark with the ultrasonic field. A) Spark under no ultrasonic field. B) Ultrasonic field guiding the spark, the led is used for timing purposes. C) Reaction time depending on the modulation frequency of the spark, (ON) switching on the ultrasonic field to guide the spark, and (OFF) time that takes the guidance to stop when the ultrasonic field is switched off.

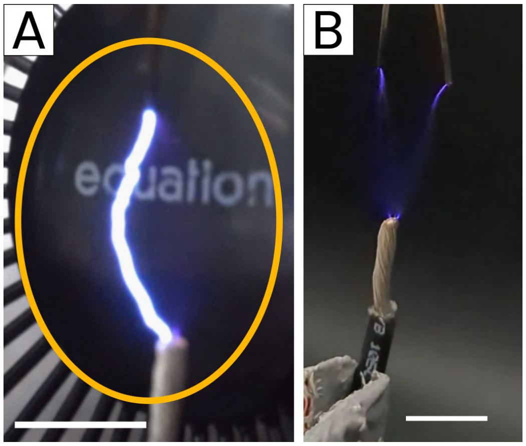

Fig. S12: A DC spark of 60 kV generated with a 5 kV step-up transformer connected to a 9-stage Cockcroft-Walton Multiplier. A) the spark was created between the positive and a ground terminal on top separated by 2 cm. the ring of ultrasonic emitters was placed around the spark to guide or push it. B) the ultrasonic field was used to make the spark go to only one of the two ground terminals on top. Scale bar is 1 cm.

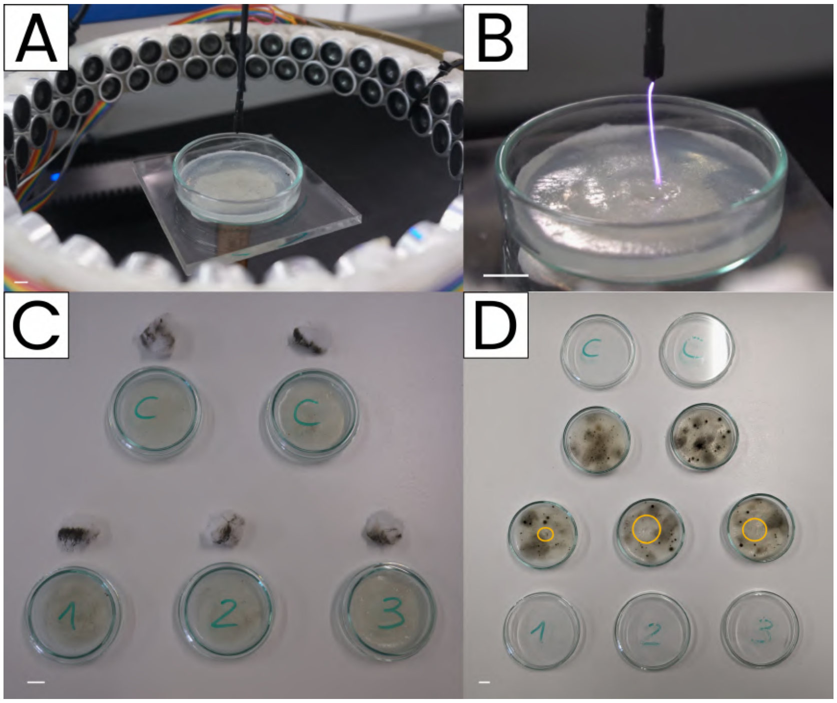

Fig. S13: Selective bacteria sterilisation. A) Petri dish with Agar and germs added from a window blind. B) Applying a guided spark to the sample, note that the ultrasonic guidance allows to direct the spark upside down. C) Two control samples and three targeted samples. D) Results after 2 weeks. Scale bars are 1 cm.

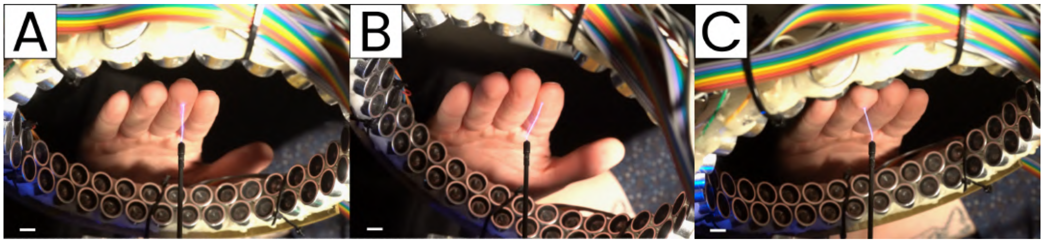

Fig. S14: Contactless Haptics: A), B) and C) guiding the spark to stimulate different fingers. Scale bar is 1 cm.

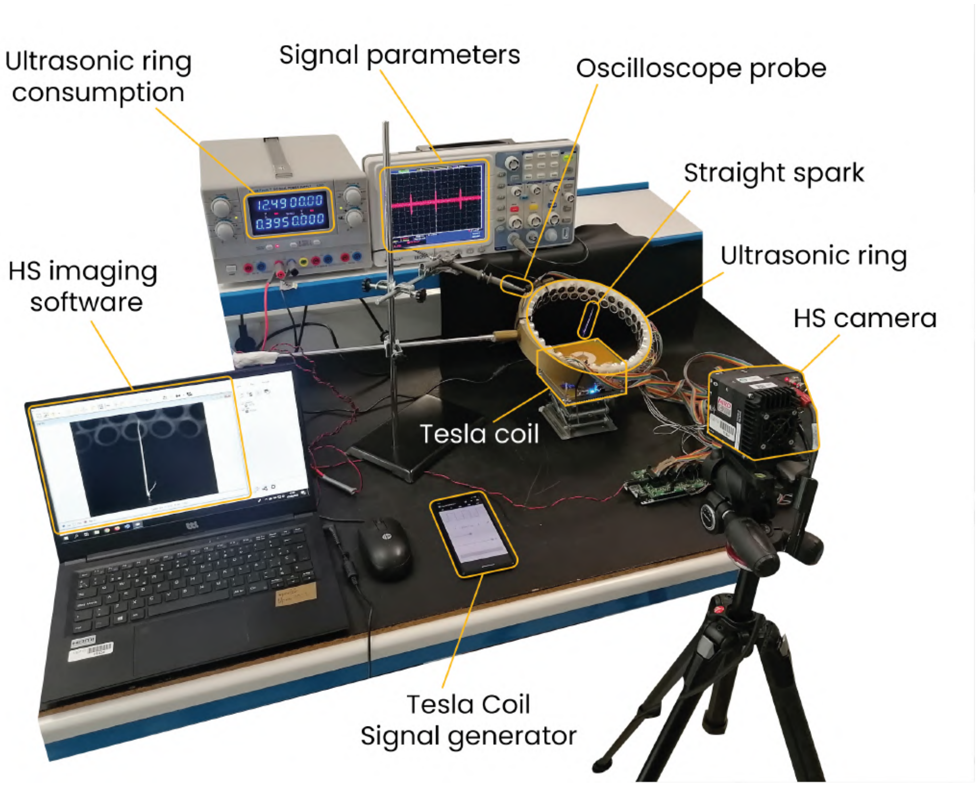

Fig. S15: Experimental setup for capturing high-speed footage.

Movie S1: Video showing the guiding effect of ultrasound on a spark, mechanical tilting, array controlled tilting, selective powering of a 3x3 array of neon bulbs, bending sparks around obstacles, and timing dynamics.

Movie S2: Video showing potential applications on haptics (tactile stimuli) targeting different fingers.

Movie S3: Animated simulations of the hot-air regions in the ultrasonic field.

Movie S4: Schilieren footage of the spark with and without ultrasonic guidance
